# Kinesiophobia, Pain, Muscle Functions, and Functional Performances among Older Persons with Low Back Pain

**DOI:** 10.1155/2017/3489617

**Published:** 2017-05-29

**Authors:** Nor Azizah Ishak, Zarina Zahari, Maria Justine

**Affiliations:** Centre for Physiotherapy, Faculty of Health Sciences, Universiti Teknologi MARA, 42300 Bandar Puncak Alam, Selangor, Malaysia

## Abstract

**Objectives:**

This study aims (1) to determine the association between kinesiophobia and pain, muscle functions, and functional performances and (2) to determine whether kinesiophobia predicts pain, muscle functions, and functional performance among older persons with low back pain (LBP).

**Methods:**

This is a correlational study, involving 63 institutionalized older persons (age = 70.98 ± 7.90 years) diagnosed with LBP. Anthropometric characteristics (BMI) and functional performances (lower limb function, balance and mobility, and hand grip strength) were measured. Muscle strength (abdominal and back muscle strength) was assessed using the Baseline® Mechanical Push/Pull Dynamometer, while muscle control (transverse abdominus and multifidus) was measured by using the Pressure Biofeedback Unit. The pain intensity and the level of kinesiophobia were measured using Numerical Rating Scale and Tampa Scale of Kinesiophobia, respectively. Data were analyzed using Pearson's correlation coefficients and multivariate linear regressions.

**Results:**

No significant correlations were found between kinesiophobia and pain and muscle functions (all *p* > 0.05). Kinesiophobia was significantly correlated with mobility and balance (*p* = 0.038, *r* = 0.263). Regressions analysis showed that kinesiophobia was a significant predictor of mobility and balance (*p* = 0.038).

**Conclusion:**

We can conclude that kinesiophobia predicted mobility and balance in older persons with LBP. Kinesiophobia should be continuously assessed in clinical settings to recognize the obstacles that may affect patient's compliance towards a rehabilitation program in older persons with LBP.

## 1. Introduction

Kinesiophobia or “fear of movement” was originally defined as a state where an individual experiences excessive, irrational, and debilitating fear of physical movement and activity as a result of a feeling of susceptibility to painful injury or reinjury [1]. In clinical settings, fear was recognized as an important aspect in patients' disability, which needs to be addressed to accomplish a successful outcome as it influences the rehabilitation strategies [2, 3]. Based on the fear-avoidance model [4], when pain is perceived as threatening, pain catastrophizing occurs, which may develop pain related fear and anxiety, in turn leading to avoidance behaviour. Avoidance behaviour is a state where an individual withdraws from performing activities such as leisure, work, and socializing, which are associated with high levels of pain, which may aggravate the painful experience. Subsequently, avoidance behaviour as an adaptation to pain in the long term would develop disuse, disability, and depression [5]. Kinesiophobia had been widely assessed in various conditions including Parkinson's disease, fibromyalgia, spinal stenosis, and low back pain (LBP) [6–9].

LBP is relatively common in older persons, and previous studies had suggested that LBP may lead to difficulty or inability in performing functional tasks in older persons, which further causes reduced mobility and balance [10–12]. Mobility is critical for older persons in maintaining their functional independence, in which those with poor mobility have higher rates of morbidity and mortality and poor quality of life [13–15]. In patients with LBP, several movements had been recognized as common to alleviate pain in the lower back such as lumbar flexion, extension, and rotation [16]. When certain movements alleviate pain, this will elicit fear and the individual tends to avoid these movements [17]. Avoiding painful movements of the lumbar joints for a long time may reduce the activity of the back and abdominal muscles, thereby decreasing their strength and control especially in those with LBP. Suri et al. [18] highlighted that impairments of lumbar muscles may potentially lead to persistent LBP and impact functional limitations or physical performance. In older persons, the progressive deterioration of the musculoskeletal systems combined with deconditioning syndrome due to LBP would affect the strength of the lower back muscles [19]. A high level of kinesiophobia, potentially, may further reduce the muscle functions in older persons with LBP.

Additionally, it is well known that functional limitations in older persons are partly due to normal aging processes [19–21], for instance, impairment during walking, squatting, climbing up and down the stairs, and performing sitting to standing actions. In addition, the presence of kinesiophobia in older persons with LBP may further deteriorate their overall functional performances such as sitting to standing actions, walking, and getting out of bed, which later affect their quality of life. Kinesiophobia leads to worsening of functional ability in older persons due to avoidance of physical activity, which in turn leads to reduced mobility and persistent pain [8]. A previous study had investigated the association between kinesiophobia and functional outcomes. In young adults, pain and movement related fear were the strongest predictors of functional performances [22]. In contrast, Vincent et al. [23] argued that fear of movement was not significantly correlated with functional measures among obese older persons with LBP. However, Vincent et al.'s study evaluated the functional measures using only SF-36 physical score, which was subjectively measured and may not be the best measure to represent the actual performances. In brief, there is no clear evidence of the influence of kinesiophobia on functional performances among older persons with LBP.

**Figure 1:**
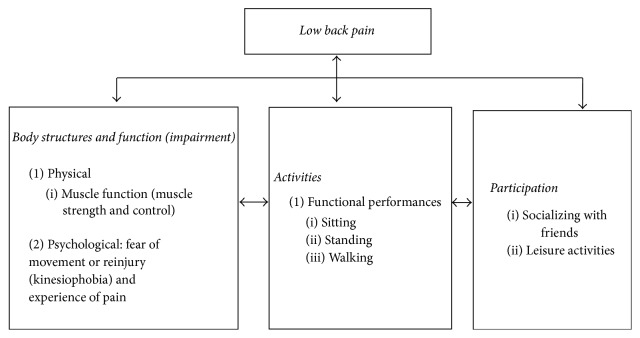
Theoretical framework based on the International Classification of Functioning, Disability and Health (ICF).

Studies had shown that older persons with LBP demonstrated high levels of kinesiophobia [16, 23]. Despite the pain and fear, advising patients to avoid painful movements or activities may not be appropriate, as this will cause further activity limitation, leading to muscle deconditioning and disuse [2, 24] which in turn might affect muscle functions and functional performance in older persons. Hence, understanding the relationships between kinesiophobia, pain, muscle functions, and functional performances is crucial in order to overcome kinesiophobia among older persons with LBP. Theoretically, the relationship between LBP, kinesiophobia, muscle functions, and functional performance can be described based on the International Classification of Functioning, Disability and Health (ICF) guidelines [25] (Figure 1). The ICF model is comprised of body structures and functions, activities, and participation. Older persons with LBP may have impairment in terms of physical functions, such as muscle functions [26], and psychological impairments such as kinesiophobia and experience of pain [27]. In addition, older persons with LBP may be limited in functional performance such as sitting, standing, and walking. Participation, such as socializing with friends and leisure activities, may also be restricted due to pain.

Although kinesiophobia had been studied in LBP subjects [5, 16, 28, 29], however, its impact on pain, muscle functions, and functional performances has not been extensively studied, especially in older persons. Therefore, this study aims (1) to determine the association between kinesiophobia and pain, muscle functions, and functional performances and (2) to determine whether kinesiophobia predicts pain, muscle functions, and functional performance among older persons with low back pain (LBP). We hypothesize that kinesiophobia is significantly correlated with pain, muscle functions, and functional performances. The outcomes of this study may provide a fundamental understanding of the interactions of kinesiophobia, pain, muscle functions, and functional performances and attributes to clinical management of kinesiophobia in older persons with LBP.

## 2. Methods

### 2.1. Participants and Study Design

This is a correlational study, involving 63 institutionalized older persons (age range = 60 to 89 years) from four selected publicly funded institutions for older persons in Malaysia. The selection of these homes was based on the preliminary survey that found that those places provided a high prevalence of older persons that complain of LBP. The participants were included in the study when the following criteria were satisfied: (1) older persons, aged 60 years and above; (2) having low back pain/backache/back pain/back disorder, diagnosed by the resident doctors; (3) being able to walk independently with or without walking aids; (4) being able to carry out activities of daily living independently; (5) being able to understand and respond to Malay/English language and able to follow instructions on testing procedures. The participants were excluded when they present with (1) permanent disability, comorbidity such as presenting with mental disorder, waiting for surgery, spinal tumour, senility, dependence most of the time, and serious spinal complication (red flags) [26] and (2) cognitive impairment (score of Mini-Mental State Examination less than 24) [29]. Ethical approval was received from the Research Ethics Committee of the Faculty of Health Sciences, Universiti Teknologi MARA (UiTM). Permission to conduct the study was received from the Social Welfare Department of Malaysia. All of the participants included in this study signed informed consent prior to the commencement of the study.

### 2.2. Outcome Measures

#### 2.2.1. Anthropometric Data

Anthropometric characteristics, including height (m), weight (kg), and BMI (kg/m^2^), were measured, in accordance with a standard protocol.

#### 2.2.2. Evaluation of Pain

The current pain intensity in the lower back region was measured using the Numerical Rating Scale (NRS). LBP is defined as pain between the costal margins and the inferior gluteal folds accompanied with limitation to perform movement [30], which was diagnosed by medical doctors. The NRS is an appropriate measure for unidimensional pain intensity, with a sensitivity of 0.68, specificity of 0.62, a standard error measurement of 1.02, and minimum detectable change of 2 points [30–32]. The NRS is a segmented numeric version of the visual analogue scale, with “0” showing no pain and “10” the worst pain, in which participants reflect a whole number (0–10 integers) that best represents their intensity of pain [32]. Higher NRS indicates the severity of LBP. In addition, the specific movements that provoked pain at the lower back were assessed, including bending forward and backward, trunk rotation, side flexion, and combination of rotation and side flexion [16]. The specific activities that might elicit LBP such as sitting to standing, climbing up and down the stairs, walking, squatting, crossing over obstacles, lifting objects, reaching overhead objects, reaching objects on the floor, prolonged lying, prolonged sitting, and prolonged standing were also examined. Because kinesiophobia might be predisposed by the existence of other joint pain, for instance, neck, shoulder, elbow, wrist and hand, hip, knee, and the ankle or foot, therefore, we asked each participant about the presence of other joint pain [16]. The other joint pain was recorded if participants complain of pain in the neck, shoulder, elbow, wrist and hand, hip, knee, and the ankle or foot during the assessment.

#### 2.2.3. Muscle Functions

*(1) Back and Abdominal Muscle Strength*. The abdominal and back muscle strength was evaluated using a Baseline Mechanical Push/Pull Dynamometer (MPPD) 22 lb (Fabrication Enterprises Inc., USA). The abdominal and back muscles are significant to be assessed as these muscles were superficial muscles that create and control the movement of the trunk [33]. The test-retest reliability of the Push/Pull Dynamometer was acceptable with intraclass correlations (ICCs) value from 0.85 to 0.99 [34]. For the testing of back muscle strength, participants were positioned in a prone lying position with the MPPD placed along the lumbar spine. The participants were instructed to lift up their body against the device [35]. For abdominal muscle strength testing, participants were asked to lift up their body against the MPPD that was placed two inches below the xiphoid process in a crook lying position [35]. Participants were required to perform isometric contractions for 4 seconds with three repetitions with 30-second rest in every trial for both tests. The procedures were repeated three times and the average reading was calculated in each test. Participants were allowed to practice the test before the actual measurement was taken. The unit of reading for MPPD is in kilogram and higher score represents a stronger back and abdominal muscle.

*(2) Muscle Control*. The muscle control of the transverse abdominus (TrA) and multifidus was measured by using the Pressure Biofeedback Unit (PBU). The TrA and multifidus are important as they increase the intra-abdominal pressure that is responsible for the stability of the spine [36]. The reliability study of PBU test demonstrated ICCs of 0.81 for the test-retest reliability [37]. The clinimetric analysis of PBU test showed that this test had low sensitivity of 0.22, moderate specificity of 0.77, a positive likelihood ratio of 0.94, and a negative likelihood ratio of 1.02 [38]. The testing procedures were as follows: participants were asked to draw in their abdomen without moving the spine or pelvis and hold for 10 seconds in a prone lying position [39]. In the prone lying position, the inflatable bag of the PBU was placed between the anterior superior iliac spine and navel. The pressure for PBU was set at 70 mmHg, and the pressure reduction readings were recorded. For the testing of multifidus muscle control, participants were instructed to draw in their abdomen and hold for 10 seconds in a crook lying position. In the crook lying position, the inflatable bag of the PBU was placed along the lumbar spine. The pressure for PBU was set at 40 mmHg, and the readings of the pressure reduction were recorded. For both tests, participants were allowed to train and practice before the actual test. The pressure reduction from 0 to 3 mmHg and 0 to 2 mmHg indicates good and fair muscle control, respectively, and an increased pressure from the initial pressure indicates poor muscle control [40].

#### 2.2.4. Functional Performances

*(1) Lower Limb Function*. The 30-second chair rise test was used to evaluate lower limb function, which is needed in day-to-day activities, such as getting out of a chair or climbing stairs. The 30-second chair rise test had acceptable level of interrater reliability (ICC = 1), sensitivity (66.7%), and specificity (67.9%) [41, 42]. The 30-second chair rise test is a valid and reliable tool in assessing functional strength and endurance in the lower extremities in older adults [43]. This test required repetitive standing up and sitting down movements, in which patients with LBP might have difficulties in performing. In this test, participants were instructed to stand upright from a chair, sit down again, and repeat the task in 30 seconds [44]. The cut-off point for this test is 15 repetitions [45], and numerous repetitions of sitting to standing represent a good lower limb function. 

*(2) Mobility and Balance*. The timed up-and-go (TUG) test was used to assess balance and mobility among participants. This test has been widely used in assessing functional mobility and balance in various conditions such as osteoporosis, lumbar degenerative disease, and musculoskeletal problems [46–48]. This test is relatively quick and simple, which examines the speed of functional balance and mobility, such as standing, walking, turning tasks, and sitting down, which might be difficult for older persons with LBP. TUG test had high interrater reliability ICC = 0.98 [49, 50] for the assessment of functional mobility. The TUG test also had 73.7% sensitivity and 65.8% specificity for the predictive value of discriminating older persons who fell at the cut-off values of 12.47 [51]. In this test, participants were seated on a chair (approximately 46 cm) and were required to stand up, walk a 3-meter distance at a normal pace, turn, walk back, and sit again [50]. The cut-off time for the TUG test is 13.5 seconds. The TUG time above 13.5 seconds indicates poor mobility in older persons [52]. 

*(3) Hand Function*. The hand function was assessed via measuring the hand grip strength of participants, using a handheld dynamometer. This test demonstrated an acceptable level of validity in measuring hand grip strength [53]. This test is easy and useful in identifying the decline of functional performances. This test reflects the overall functional performances in older persons which may decrease, especially in those with LBP. In this test, participants were positioned in a sitting position while gripping the dynamometer with elbow in 90-degree flexion, with the forearm and hand in a neutral position. The testing procedure [54] required participants to squeeze the handle of the dynamometer as strong as they can. The measurements were taken for the dominant hand with one-minute rest in between each attempt. The cut-off value for hand grip strength is 30 kg for males and 20 kg for females [55], and a higher score indicates a greater hand grip strength.

#### 2.2.5. TAMPA Scale of Kinesiophobia

The Tampa Scale of Kinesiophobia-11 (TSK-11) was used to measure the level of fear of movement or reinjury. The original versions of this questionnaire had an acceptable level of internal consistency (Cronbach's *α* of 0.8), evidence of discriminants, and concurrent criterion related and incremental validity [56]. The TSK-11 consists of 11 questions that can be divided into two factors which are somatic factors and activity avoidance [57]. The somatic focus would predict perceived disability and activity avoidance focus on actual physical performance, controlling for pain severity [58]. In this study, the Malay® version of TSK-11 was used, with an acceptable level of internal consistency (Cronbach's *α* of 0.84) and test-retest reliability (ICC = 0.87) [59]. This outcome measure consists of 11 items and each item was scored based on a 4-point Likert scale, ranging from “strongly disagree” to “strongly agree.” The scoring of TSK-11 ranged from 11 to 44, in which a higher score of TSK-11 indicates a higher level of kinesiophobia. Since the TSK questionnaire does not have items related to fear of back specific movements, the movements that might induce fear were assessed, including bending forward and backward, rotation, and side flexion of the trunk. The specific activities that may lead to kinesiophobia such as sitting to standing, climbing up and down the stairs, walking, squatting, crossing over obstacles, lifting objects, reaching overhead objects, reaching objects on the floor, prolonged lying, prolonged sitting, and prolonged standing were evaluated.

### 2.3. Statistical Analysis

The IBM SPSS statistical software version 20 was used to conduct descriptive statistics, correlation, and regression analyses. The mean and standard deviation of all the variables were calculated and the significance level was set as *p* < 0.05 for each of the statistical analyses. Power analysis was conducted using G-Power 3 software© [60], where power is set at 0.8 and *α* at 0.05 using Correlation: Point Biserial Model. Therefore, the sample size of 63 participants was sufficient to provide moderate effect for the correlation analysis. Pearson's correlation coefficient was used to determine the association between kinesiophobia and pain, muscle functions, and functional performances and was interpreted as follows: less than 0.3 (poor), 0.3 to 0.5 (fair), 0.6 to 0.8 (moderately strong), and 0.8 and above (very strong) [61]. In addition, multivariate linear regression analysis was conducted to determine whether kinesiophobia predicts pain, muscle functions, and functional performance. The outliers whose scores were out of the score range that largely influences statistical analysis were excluded manually.

### 2.4. Characteristics of the Participants

Table 1 shows the characteristics (age and BMI), pain intensity, functional performances (lower limb function, balance and mobility, and hand grip strength), muscle functions (abdominal and back muscle strength, TrA and multifidus muscle control), TSK-11, and duration of LBP among groups of 63 older persons involved in this study. 60.3% of participants in this study complained of LBP for more than 6 months.

Table 2 demonstrates the presence of joint pain at other sites and movements and activities inducing LBP and kinesiophobia. Most of the participants complained from pain in the knee (39.7%), shoulder (25.4%), and foot and ankle (19%). Bending trunk was identified as the most frequent movement that induced pain in the lower back (46%). In addition, for the specific activities, sitting to standing and prolonged sitting were noted as the top activities that trigger LBP (44.4%), followed by walking and lifting objects (31.7%).

It is interesting to note that most participants in this study had a higher percentage of self-reported kinesiophobia (52.4%). In addition, trunk flexion and side flexion were identified as the most frequent movements that trigger kinesiophobia (15.9%). For the specific activities that increase fear, the most frequent activity that increases kinesiophobia was the sitting to standing movement (23.8%). Another two activities that induced kinesiophobia were prolonged standing (22.2%) and walking and prolonged sitting (20.6%).

### 2.5. Correlation of Kinesiophobia, Pain, and Muscle Functions

Table 3 indicates the correlation between kinesiophobia and pain and muscle functions. Contrary to our expectations, the results showed that kinesiophobia was not correlated with pain intensity (*p* > 0.05). In addition, there was also no significant correlation between kinesiophobia and all variables of muscle functions (all *p* > 0.05).  Table 4 shows the analysis using multivariate linear regression between kinesiophobia, pain, and muscle functions. Kinesiophobia was not a significant predictor of pain and muscle functions in older persons with LBP (all *p* > 0.05).

### 2.6. Correlation of Kinesiophobia and Functional Performance

As in Table 3, an important finding was that kinesiophobia showed significant correlation with mobility and balance (*p* = 0.038). However, kinesiophobia did not show any significant correlation with other functional performance variables which were hand grip strength and lower limb function (*p* = 0.74 and *p* = 0.125, resp.). The result of multivariate linear regression between kinesiophobia and functional performance as indicated in Table 4 demonstrated that kinesiophobia predicted mobility and balance (*p* = 0.038) in older persons with LBP. However, kinesiophobia was not a significant predictor of hand grip strength (*p* = 0.740) and lower limb function (*p* = 0.125).

## 3. Discussion

### 3.1. The Correlation between Kinesiophobia, Pain, and Muscle Function

This study aimed to determine the correlation between kinesiophobia, pain, muscle functions, and functional performances in older persons with LBP. Our study supplements an important dimension to the findings of research on LBP in older persons.

Previous studies had found a significant correlation between kinesiophobia and pain intensity in older persons with LBP [5, 16, 58]. However, our study did not demonstrate similar findings, as we found insignificant correlation between kinesiophobia and pain in older persons with LBP. The unexpected findings in our study could be due to the moderate pain level in the participants, which is 4.17 only. Besides, the participants scored 29.67 in the TSK questionnaire, and Larsson et al. [5] had classified TSK greater than 35 as high levels of kinesiophobia. Therefore, it can be generalized that participants in this study had moderate levels of kinesiophobia and pain. In addition, resilience, which is a positive personality that enhances adaptation to threats [62], possibly exists among participants in the current study. The majority of the participants in our study were older persons with chronic LBP, and studies had shown that optimism and high resilience were related to the reduction of pain intensity and pain catastrophizing in chronic pain participants [63, 64]. Thus, this might explain the insignificant correlation between kinesiophobia and pain in the study.

The present study revealed that kinesiophobia did not correlate with back and abdominal muscle strength. These findings did not seem to fit with our hypothesis suggesting that muscle function is linked with fear of movement. However, our study is in agreement with Demoulin et al.'s [65] findings, which showed that related fear measure was not significantly correlated with back muscle strength. It is difficult to explain this result, but it might be related to the moderate total score of TSK. In addition, the testing procedures of back and abdominal muscle strength were conducted in a supine lying position, whereby the testing is stable and may not be compatible with the level of fear to movements among older persons with LBP. In the future, back and abdominal muscle strength tests should be conducted in a functional task that may induce fear such as lifting task.

This current study found that kinesiophobia was not correlated with muscle control of the TrA and multifidus. However, our findings were inconsistent with Massé-Alarie et al. [66] that revealed that kinesiophobia was significantly correlated with overactivation of TrA during forward bending trunk movements, indicating the possible influence of kinesiophobia on TrA muscle control. The unexpected findings might be explained in this way. In our study, the muscle control of the TrA and multifidus was tested in dynamic conditions of testing, which were prone and supine lying positions, respectively. The tests were conducted in relaxing, stable, and pain-free positions; thus, the fear of movements might not exist. By contrast, Massé-Alarie et al. used electromyography to test the activation of TrA during trunk flexion, whereby the activation of TrA was peaked during the onset of extension and at the end of trunk flexion during the trunk flexion task. The authors also stated that, during full flexion, the position of the spine was close to the body, whereby the posterior passive tissues are in the stress condition, thus increased kinesiophobia in LBP as the position in pain and fear are frequently felt. In addition, George et al. [67] highlighted the notion that patients with acute or subacute LBP had a fear of loaded spine activities, postural components, and specific spinal motions. Therefore, in future studies, the assessment of TrA and multifidus using PBU should be evaluated in movements or activities that induce fear, to provide different results and a better understanding of the influence of kinesiophobia on the muscle control of TrA and multifidus.

### 3.2. The Correlation between Kinesiophobia and Functional Performances

Pain experience initiates kinesiophobia which later leads to avoidance behaviour that may affect functional performances including lower limb functions. Conversely, in our study, we discovered no significant correlation between kinesiophobia and lower limb function among older persons with LBP. Despite the decline in lower limb function, which might be secondary to LBP, however, resilience possibly exists. Therefore, older persons might be able to adjust the difficulties to perform activities involving lower limb function, neglect the fear, and continue normal daily activities. In our study, only 23.8% of the participants reported fear during sitting to standing tasks, showing that they are not that fearful to do the respective task. In addition, our participants lived in the institutions, where they received social support such as emotional, informational, and companionship support from their friends, staffs, and volunteers. Wells [68] reported that strong social ties are associated with resilience. Therefore, the weak correlation between kinesiophobia and lower limb function among older persons with LBP can be assumed due to the resilience factor.

It is noteworthy that we discovered a significant and moderate correlation between mobility and balance with kinesiophobia among older persons with LBP. Kinesiophobia also was a significant predictor of mobility and balance. The finding of the current study is consistent with a previous study [23], which found that TSK was associated with walking impairment. However, Vincent et al. used a different method of assessing walking impairment, as they performed measurements by a subscale in the Oswestry Disability Index Questionnaire. This test seems to be a subjective measure and does not truly represent the participants' actual walking ability and their mobility. The participants may also rate their mobility and walking ability inaccurately and thus this may affect the results. Contrary to the current finding, we used an objective measure of mobility assessment which was TUG, which seems to be accurate [51]. The influence of kinesiophobia on mobility and balance in older persons with LBP seems to be obvious if it is measured by objective testing. Despite the different methods used, it can be generalized that kinesiophobia was associated with and may influence mobility and balance in older persons with LBP.

A previous study [69] revealed that kinesiophobia and catastrophizing thinking were the main predictors of the upper extremity-specific disability, which is significantly associated with hand grip strength [70–72]. However, in our study, kinesiophobia was not correlated with hand function. The unexpected finding might be explained in this way. Despite the functional decline of hand grip strength among older persons with LBP, however, there is a possibility of adaptation to pain as subjects need to carry out activities of daily living involving hand movement independently such as dressing, eating, and bathing. Furthermore, only 17.5% of the participants reported wrist or hand pain, in which the pain is not affecting them much rather than LBP. In addition, the somatic focus score among participants reflects the belief of underlying and serious problems of the back region but not the hand region, which might explain why kinesiophobia was not associated with hand grip strength.

### 3.3. Study Limitations

We determined several limitations in this study. Firstly, the study has been designed based on a correlational study with 63 participants, in which the sample size is relatively small and the findings cannot be generalized to a whole population of older persons. Subsequently, due to the small sample size, we combined the correlational and regression analyses of both male and female older persons, in which their characteristics in terms of muscle functions and functional performance might be different. Further study is warranted for gender comparison to find out any possible differences of association between kinesiophobia and pain, muscle functions, and functional performance between male and female older persons with LBP. For muscle function test, we conducted the test in supine and prone lying positions, which were not functional movement tasks that could potentially be more “fear inducing” to older persons with LBP. Therefore, it may not best reflect the influence of kinesiophobia on muscle functions. In addition, for functional performance, only hand grip strength, TUG, and 30-second chair rise test were measured. It is best if other outcome measures such as speed test, walking endurance, and back endurance can be measured to provide holistic findings of functional performance.

Besides, the muscle functions and functional performance status in older persons might be a consequence of normal aging changes, due to decline of muscle mass and also its strength, which would impair their muscle functions and functional performances. Therefore, kinesiophobia might not be the main factor that determines muscle functions and functional performances in older persons, as normal aging changes are likely rather an important factor. Last but not least, our study recruited samples from four selected publicly funded institutions, making our study at high risk of selection bias.

Despite these limitations, to the best of the authors' knowledge, this paper presents the first study that evaluated the association between kinesiophobia and pain, muscle functions, and functional performances among older persons with LBP. Although the results in our study did not reach statistical significance, our study adds to the new understanding of the interaction between kinesiophobia, pain, muscle functions, and functional performances in older persons with LBP. Further study is needed to provide a broader understanding of these interactions, perhaps with different measures. We suggest conducting a similar study with a larger sample size and other functional tests to allow for more accurate evaluation between the variables.

## 4. Conclusion

In conclusion, our study demonstrated that kinesiophobia was not associated with pain and muscle functions in older persons with LBP. Kinesiophobia was associated with mobility and balance but not with lower limb function and hand grip strength. Kinesiophobia also only predicted mobility and balance but not other variables of functional performance. Therefore, the association of kinesiophobia, pain, muscle functions, and functional performances in older persons with LBP should be investigated in the future, possibly by exploring other outcomes, to further validate the current findings. Kinesiophobia should be continuously assessed in clinical settings to recognize the obstacles that may affect patient's compliance towards a rehabilitation program in older persons with LBP.

## Figures and Tables

**Table 1:** Characteristics of the participants (*n* = 63).

Variables	Mean ± SD (range)
Age (years)	70.98 ± 7.90 (60–89)
BMI (kg/m^2^)	23.70 ± 4.18 (16.4–37.1)
Pain intensity	4.17 ± 1.70 (1–9)
Abdominal strength (kg)	0.34 ± 0.06 (0.20–0.46)
Back strength (kg)	0.33 ± 0.05 (0.23–0.45)
TrA control (mmHg)	69.14 ± 3.00 (63.3–75.34)
Multifidus control (mmHg)	40.45 ± 2.38 (35–46)
Lower limb function (reps)	9.35 ± 3.18 (3–17.67)
TUG (s)	13.38 ± 4.81 (6.08–25.89)
Hand grip strength (kg)	17.51 ± 7.20 (5–36)
TSK total score	29.67 ± 7.92 (11–44)
Somatic focus	13.89 ± 4.32 (5–20)
Avoidance activity	16.00 ± 4.33 (6–24)
Duration of LBP	*n* (%)
<1 month	11 (17.5)
1–3 months	10 (15.9)
3–6 months	4 (6.3)
>6 months	38 (60.3)

**Table 2:** Presence of joint pain at other sites and movements and activities inducing LBP and kinesiophobia.

	Yes (%)	No (%)
Do you have pain in the following sites?		
Neck	7 (11.1)	56 (88.9)
Elbow	6 (9.5)	57 (90.5)
Hip	8 (12.7)	55 (87.3)
Foot or ankle	12 (19.0)	51 (81.0)
Shoulder	16 (25.4)	47 (74.6)
Wrist of hand	11 (17.5)	52 (82.5)
Knee	25 (39.7)	38 (60.3)
Do these movements increase your back pain?		
Trunk rotation	10 (15.9)	53 (84.1)
Bending backward	7 (11.1)	56 (88.9)
Side flexion	5 (7.9)	58 (92.1)
Bending trunk	29 (46)	34 (54)
Side flexion and rotation	12 (19)	51 (81)
Do these activities increase your back pain?		
Sitting to standing	28 (44.4)	35 (55.6)
Climb up and down the stairs	16 (25.4)	47 (74.6)
Walking	20 (31.7)	43 (68.3)
Squatting	14 (22.2)	49 (77.8)
Cross over an obstacle	7 (11.1)	56 (88.9)
Lifting objects	20 (31.7)	43 (68.3)
Reach overhead objects	8 (12.7)	55 (87.3)
Reach objects on the floor	7 (11.1)	56 (88.9)
Prolonged lying	7 (11.1)	56 (88.9)
Prolonged sitting	28 (44.4)	35 (55.6)
Prolonged standing	19 (30.2)	44 (69.8)
Self-reported kinesiophobia	33 (52.4)	30 (47.6)
Do these movements increase your fear?		
Trunk rotation	6 (9.5)	57 (90.5)
Bending backward	4 (6.3)	59 (93.7)
Side flexion	10 (15.9)	53 (84.1)
Bending trunk	10 (15.9)	53 (84.1)
Side flexion and rotation	9 (14.3)	54 (85.7)
Do these daily activities increase your fear?		
Sitting to standing	15 (23.8)	48 (76.2)
Climb up and down the stairs	9 (14.3)	54 (85.7)
Walking	13 (20.6)	50 (79.4)
Squatting	7 (11.1)	56 (88.9)
Cross over an obstacle	3 (4.8)	60 (95.2)
Lifting objects	11 (17.5)	52 (82.5)
Reach overhead objects	2 (3.2)	61 (96.8)
Reach objects below	4 (6.3)	59 (93.7)
Prolonged lying	3 (4.8)	60 (95.2)
Prolonged sitting	13 (20.6)	50 (79.4)
Prolonged standing	14 (22.2)	49 (777.8)

**Table 3:** Pearson's correlation coefficient between kinesiophobia, pain, muscle functions, and functional performances (*n* = 63).

	Correlates	Kinesiophobia *r* *p* value
Pain	Pain intensity	0.129
		0.314

Muscle functions	Abdominal strength	0.126
		0.327
	Back strength	0.079
		0.537
	TrA control	0.050
		0.694
	Multifidus control	0.156
		0.222

Functional performances	Lower limb function	−0.195
		0.125
	TUG	**0**.263^**∗**^
		**0.038**
	Hand grip strength	0.043
		0.740

Correlation was tested using Pearson's correlation coefficient analysis. ^*∗*^Correlation is significant at the level of 0.05 (1-tailed).

**Table 4:** Multivariate linear regression of TSK and explanatory variables (*n* = 63).

Variable	B	SE	*p* value
Pain intensity	0.028	0.027	0.314
Abdominal strength	0.001	0.001	0.327
Back strength	0.000	0.001	0.537
TrA control	0.019	0.048	0.694
Multifidus control	0.047	0.038	0.222
Lower limb function	−0.079	0.050	0.125
TUG	0.159	0.075	**0.**038^**∗**^
Hand grip strength	0.039	0.116	0.740

Test was conducted using multivariate linear regression. ^*∗*^The  *p* value is significant at the level of 0.05 (1-tailed).
